# Linkage Disequilibrium, Haplotype Block Structures, Effective Population Size and Genome-Wide Signatures of Selection of Two Conservation Herds of the South African Nguni Cattle

**DOI:** 10.3390/ani12162133

**Published:** 2022-08-19

**Authors:** Njabulo M. Dlamini, Edgar F. Dzomba, Mpumelelo Magawana, Sphamandla Ngcamu, Farai C. Muchadeyi

**Affiliations:** 1 Discipline of Genetics, School of Life Sciences, University of KwaZulu-Natal, Private Bag X01, Scottsville, Pietermaritzburg 3209, South Africa or; 2 Agricultural Research Council, Biotechnology Platform, Private Bag X5, Onderstepoort, Pretoria 0110, South Africa; 3 KZN Department of Agriculture & Rural Development, Private Bag X9059, Pietermaritzburg 3200, South Africa

**Keywords:** linkage disequilibrium, haplotype blocks, effective population size, selection signatures, conservation herds

## Abstract

**Simple Summary:**

Knowledge of linkage disequilibrium (LD), haplotypes blocks, and selective sweeps is important for effective application of genomics in breed characterization, improvement, and conservation, amongst other uses. The South African Nguni cattle breed is a Sanga breed that is well known for its ability to adapt to various environmental conditions such as harsh pedoclimatic and socio-economic conditions which exist in semiarid areas. Nguni cattle are characterized by many eco-types and research populations have been established in an effort to conserve the diversity within the breed by sampling in animals from different ecotypes into the herds. In this study, we calculated autosomal linkage disequilibrium, haplotype block structure, and screened for selection sweeps in two Nguni conservation herds of Bartlow Combine (*n* = 85) and Kokstad (*n* = 42), whose animals were genotyped on the Illumina High-Density Bovine SNP BeadChip^®^. The two herds were generally similar based on a number of genetic parameters. Overall, the study implied reduced genetic diversity in the two herds, calling for corrective measures to maintain the diversity of the South African Nguni cattle.

**Abstract:**

The Nguni cattle of South Africa are a Sanga breed, characterized by many eco-types and research populations that have been established in an effort to conserve the diversity within the breed. The aim of this study was to investigate the overall genetic diversity as well as similarities and differences within and between two conservation herds of the South African Nguni Cattle. Mean LD (*r*^2^) estimates were 0.413 ± 0.219 for Bartlow Combine and 0.402 ± 0.209 for Kokstad. Genome-wide average LD (*r*^2^) decreased with increasing genetic marker distance for both populations from an average of 0.76 ± 0.28 and 0.77 ± 0.27 at 0–1 kb bin to 0.31 ± 0.13 and 0.32 ± 0.13 at 900–1000 kb bin in Bartlow Combine and Kokstad populations, respectively. Variation in LD levels across autosomes was observed in both populations. The results showed higher levels of LD than previously reported in Nguni field populations and other South African breeds, especially at shorter marker distances of less than 20 kb. A total number of 77,305 and 66,237 haplotype blocks covering a total of 1570.09 Mb (61.99% genome coverage) and 1367.42 Mb (53.96% genome coverage) were detected in Bartlow Combine and Kokstad populations, respectively. A total of 18,449 haploblocks were shared between the two populations while 58,856 and 47,788 haploblocks were unique to Bartlow Combine and Kokstad populations, respectively. Effective population size (N_e_) results demonstrated a rapid decrease in N_e_ across generations for both Bartlow Combine and Kokstad conservation herds. Two complementary methods, integrated haplotype score (iHS) and Extend Haplotype Homozygosity Test (XP-EHH), were implemented in this study to detect the selection signatures in the two herds. A total of 553 and 166 selected regions were identified in Bartlow Combine and Kokstad populations, respectively. DAVID and GO terms analysis of the regions under selection reported genes/QTLs associated with fertility, carcass weight, coat colour, immune response, and eye area pigmentation. Some genes, such as *HCAR1, GNAI1, PIK3R3, WNT3, RAB5A, BOLA-N* (Class IB *MHC* Antigen QA-2-Related), *BOLA* (Class *IB MHC* Antigen QA-2-Related), and *Rab-8B,* etc., were found in regions under selection in this study. Overall, the study implied reduced genetic diversity in the two herds calling for corrective measures to maintain the diversity of the South African Nguni cattle. This study presented a comprehensive analysis of the genomic architecture of South African Nguni cattle populations, providing essential genetic information of utility in the management of conservation flocks.

## 1. Introduction

The Nguni (Sanga type) cattle breed is one of the major indigenous cattle breeds that is hardy and is uniquely adapted to different ecological regions of South Africa [1]. Migration, genetic drift, and selection all played an important role in the Nguni cattle breed’s early development [2]. Along with environmental adaptation, breed hybridization has contributed to the array of coat colour phenotypes observed among Nguni cattle populations [2]. The Nguni indigenous cattle of South Africa are kept in different geographical regions of the country, where they have adapted to the various environmental conditions [3]. Some important traits reported in the Nguni cattle include resistance to local diseases and parasites [4,5,6], adaptation to low-quality feed resources [7], and heat tolerance [8].

Indigenous breeds such as the Nguni are not well characterized or described and are rarely subjected to structured breeding efforts to improve their performance [9]. More importantly, due to uncontrolled crossbreeding and institutional policies that encourage the use of high-producing exotic breeds in the smallholder regions, these indigenous animal genetic resources are in constant decline [10,11]. Pure Nguni cattle populations in South Africa decreased from 1,800,000 in 1992 to 9462 in 2003, putting the purity of indigenous cattle breeds in danger [12]. The unrestricted introduction of exotic genotypes into indigenous herds has resulted in a decrease in pure Nguni cattle in South Africa [13].

The steady deterioration of the Nguni gene pool will ultimately result in the loss of vital genes that have allowed the Nguni to thrive and produce for generations under the country’s extreme environmental conditions [14]. In recent years, there has been a surge in interest in reviving and preserving indigenous breeds due to their ability to adapt to harsh environmental conditions and their importance as a source of genetic variety. Nguni research populations such as those at Bartlow Combine Research Station and Kokstad Research Station in KwaZulu-Natal region of South Africa were established in an effort to maintain and conserve nucleus of pure-bred Nguni cattle [15]. Conservation and research populations are often accompanied with the risk of (i) losing the required level of diversity and (ii) divergence from the breed attributes. The characterization of the conservation herds of Nguni cattle in Bartlow Combine and the Kokstad research stations is a step necessary in ensuring that both the overall genetic diversity and unique genetic attributes of the herds are established and conserved.

Linkage disequilibrium (LD), effective population size (N_e_), and signatures of selection are key genomic parameters that can be used to assess genetic diversity, regions under positive selection, and to determine whether the conservation populations are viable or at another risk of extinction. Haplotype block structures are characterized as areas with a high marker–marker LD and a low haplotype diversity separated by short regions of very low LD [16]. The identification of haplotype blocks can transform information on several SNPs into haplotype block information [17].

Altogether, characterized haplotype blocks and LD patterns may provide useful tools for gaining insight into economically relevant genetic effects of selection and other evolutionary processes acting on breed genomes, as well as the population’s overall genetic viability [18,19]. The LD pattern is a powerful indicator of the genetic processes driving a population and understanding LD may help in inferring a population’s N_e_ and historical demographics [20]. Historical N_e_ estimates reveal important demographic features, such as population growth rates and the occurrence of bottleneck events in the past [21,22].

The detection of selection signatures has been a popular concept in recent years because of its ability to uncover genes and advantageous mutations associated with ecologically and economically important traits [23]. Furthermore, the detection of selection signatures may be utilized in conjunction with genome-wide association studies (GWAS) to associate candidate genes under selection with the phenotypes, which can then be implemented in genomic selection and assisted breeding [24,25].

The objectives of this study were to (i) investigate LD, haploblock patterns, and Ne of conservation herds of Nguni cattle kept in two research stations of Bartlow Combine and Kokstad and (ii) screen for selection footprints within and between the genomes of the two conservation herds. Overall, the study aimed to use differences in LD levels, haploblock structures, and regions under selection in these two conservation Nguni populations to make inferences on the diversity within and between the herds and evolutionary changes in the population sizes and genomic architecture of the conserved animals.

## 2. Materials and Methods

### 2.1. Populations and Samples

A total number of 143 Nguni blood samples were collected from two research herds, one in Kokstad (44) and another one in Bartlow Combine (99), in KwaZulu-Natal Province, South Africa. Bartlow Combine is one of the six Agricultural Research Stations in KwaZulu-Natal that was established in 1954 and is situated within the Umkhanyakude District Municipality, 40 km from Hluhluwe and 46 km from Mkuze (27°54′ S, 32°03′ E; rainfall ranges from 605 mm in the lowveld to 710 mm in the thornveld) [26]. Cattle comprising the Bartlow Combine herd were bought from local Zulu people living in the vicinities of Nongoma, Ingwavuma, Ubombo, and Hlabisa in KwaZulu-natal and were established to maintain a nucleus of pure-bred Nguni cattle [27].

Kokstad Research Station is situated in the Harry Gwala District Municipality, approximately 5 km out of Kokstad on the road to Franklin (S30°31′ 16 72, E29°24′ 30 38; rainfall: 750 mm/annum). The station, which is roughly 1200 hectares in size, was established in 1962 from a combination of town commonage and State Forestry Reserve [28]. The animals used in this study were reared in completely different environments and are indigenous to South Africa. These two herds are kept as conservation flocks for the Nguni cattle. The cattle in both herds presented diversity in coat colour patterns that is expected in Nguni breed.

### 2.2. Genotyping and Quality Control

Genomic DNA was extracted from whole blood samples using the DNA isolation NucleoMag^®^ VET kit (Nu-cleoMag—MACHEREY-NAGEL GmbH & Co KG, Düren, Germany) based on the manufacturer’s protocol. The quantity and quality of extracted DNA was assessed using the Qubit. The extracted samples were visualized using Ethidium bromide-based agarose gel electrophoresis. High-quality DNA samples (≥50 ng/μL) were genotyped at the Agricultural Research Council Biotechnology Platform (ARC-BTP) using the Illumina^®^ BovineHD Genotyping BeadChip (Illumina Inc., San Diego, CA, USA), which contained 777,692 SNPs in total, with an average gap size of 3.43 Kb and a median gap size of 2.68 Kb, evenly distributed throughout the bovine genome.

The ARS-UCD1.2/bosTau9 bovine assembly was used as a reference genome in this study. The two populations were merged and were then analyzed as a single Nguni population (overall population). Genotype calling was performed using GenomeStudio software (Illumina Inc., San Diego, CA, USA) according to the manufacturer’s protocols. The genotypes for each population were filtered for quality, using PLINK v1.90 software [29], and filtering was conducted separately for each population using the following parameters: (i) correspondence with Hardy–Weinberg Equilibrium (HWE) *p* < 0.000001; (ii) Minor Allele Frequency (MAF ≤0.02; (iii) Call rate < 90%. Furthermore, SNPs located on sex chromosomes and those with unknown chromosomal positions were also excluded from the downstream analyses.

A relatedness test was done using PLINK v1.90 [29] to establish independence among the individuals obtained in both populations. The pairwise IBD was estimated for pairs of individuals within each population. Individuals of a pair that had a *pi-hat* value greater than 0.45 were considered to be closely related, and thus, 16 individuals were removed from the analysis. One hundred and twenty-seven animals remained after QC, of which 85 and 42 animals belonged to Bartlow Combine and Kokstad populations, respectively.

### 2.3. Minor Allele Frequency

The PLINK v1.90 [29] was utilized to calculate the minor allele frequency (MAF) for each SNP in the studied populations after quality control measures were applied to the data. In-house RStudio software [30] R-scripts were used to analyze the distribution of allelic frequencies and were summarized as the proportion of the SNPs represented in five different MAF bins: ≥0.02 to <0.1, ≥0.1 to <0.2, ≥0.2 to <0.3, ≥0.3 to <0.4, and ≥0.4 to ≤0.5. MAF values that fell less than 0.02 were eliminated. The results were plotted for comparisons between the two Nguni herds.

### 2.4. PCA Analysis

A principal component analysis (PCA) was performed to illustrate the relationship between the Kokstad and Bartlow Combine Nguni cattle populations using RStudio software version 1.4.1106 [30].

### 2.5. Linkage Disequilibrium Estimation

Pearson’s squared correlation coefficient (*r*^2^) was used to calculate the LD between each pair of genetic markers [31]. According to Ardlie et al. [32] and Zhao et al. [33], *r*^2^ statistic is less sensitive to allelic frequencies and is more suitable for biallelic markers and allows the user to compare estimations with previous studies. Pairwise *r*^2^ values between adjacent SNPs were estimated for each autosome, and the genome-wide LD over all autosomes was estimated in each population using Plink v1.09 [29]. The *r*^2^ ranges between 0 and 1 and were calculated as follows:

(1) $$r^{2}=\frac{{\left(PABPab-PAbPaB\right)}^{2}}{PA*Pa*PB*Pb}$$

where *PA*, *Pa*, *PB*, and *Pb* are the frequencies of alleles *A*, *a*, *B*, and *b*, respectively; *PAB*, *Pab*, *PAb*, and *PaB* are the haplotype frequencies among the alleles in the population. The LD values for each Nguni subpopulation were separately estimated using the genome-wide SNP data. The PLINK commands ‘—*r*^2^—ld-window 99,999—ld-window-kb 1000—not-chr 0 x y mt—ld-window-*r*^2^ 0.2′ were applied to the 29 autosomes in order to take an interval less than 99,999 SNPs and to save in the output all SNPs pairs. R Studio software was used to calculate the average *r*^2^ values and standard deviations for each interval, as well as the LD decay [30]. The LD decay was then examined for three maximum distances between SNP pairs: 10 Kb, 100 Kb, and 1000 Kb, with SNP comparisons binned at 1 Kb, 10 Kb, and 100 Kb, respectively, for each distance. The average *r*^2^ for each bin was calculated and plotted against the inter-marker distance.

### 2.6. Haplotype Block Structure

Using the Expectation Maximization (EM) method technique implemented in PLINK v1.90 [29], the haplotype blocks were detected across autosomes within populations, using default parameters. Haploview [34] software was used to estimate haplotype block patterns for the 29 pairs of autosomal chromosomes containing SNPs at a maximum distance of 1000 kb, which by default, employs Gabriel et al.’s [35] haplotype block definition.

To eliminate spurious block formation, haplotype blocks formed by only two SNPs were removed. The unique and shared haplotype block regions within and between breeds were investigated. The overlapping block segments shared by two populations were characterized as shared haplotype blocks, and the block regions unique to each group were defined as unique haplotype blocks. An online tool [36] was used to visualize both shared and unique haplotype block regions in the genomes of Bartlow Combine and Kokstad conservation populations.

### 2.7. Effective Population Size

The SNeP program [37], which is based on the correlations between LD, N_e_, and the recombination rate, was used to estimate historical and recent N_e_ for all breeds. We utilized the default settings. Because the default maximum distance in SNeP was 4000 Kbp, N_e_ was studied, starting 50 generations ago. The equation is as follows:

(2) $$\;\;\;N_{T\left(t\right)}={\left(4\int (c_{t})\right)}^{-1}{\left(E\left[r_{adj}^{2}|c_{t}\right]\right)}^{-1}-\alpha$$

where *N_T_* = the effective population size t generations ago calculated as *t* = (2∫(c_t_)) ^−1^, c_t_ = the recombination rate; *r*^2^ adj = *r*^2^–(βn)^−1^ where *r*^2^ adj = the LD value adjusted for sample size (*n* = sample size, β = 2 when the gametic phase is known and β =1 if unknown), and *α* = a correction for the occurrence of mutations [38].

### 2.8. Detection of Selection Signatures

Two complementary haplotype-based detection approaches, integrated haplotype scores (iHS) and cross-population extended haplotype homozygozity (XP-EHH), were utilized to detect regions harbouring selection signatures within and between populations.

#### 2.8.1. Integrated Haplotype Score (iHS)

The iHS score is based on a ratio of extended haplotype homozygosities (EHH) associated with each allele [39]. The iHS statistic is applied to individual SNPs and is based on the decay of extended haplotype homozygosity (EHH), computed for ancestral (0) and derived alleles (1) at each core SNP [40]. This integrated EHH (iHH) (summed over both directions away from the core SNP) is denoted as iHHA or iHHD, depending on whether it is computed for the ancestral or derived core allele [41]. According to Voight et al. [40], the iHS score is described as within the population score for the ratio between iHHA and iHHD:

(3) $$iHS=\frac{ln\left(\frac{iHHA}{iHHD}\right)-E\left[In\left(\frac{iHHA}{iHHD}\right)\right]}{SD\left[ln\left(\frac{iHHA}{iHHD}\right)\right]}\;$$

where *iHHA* and *iHHD* represent the integrated *EHH* score for ancestral and derived core alleles, respectively. Chromosome-wise haplotype phasing was performed using fastPhase software [42]. The rehh R package v2.0.4 [43] was used to calculate |iHS| for each autosomal *SNP*.

Windows with less than 10 SNPs were removed. To determine the *p*-value at the genomic level, iHS scores for each SNP were further transformed as piHS = −log [1−2|Φ(iHS)–0.5|], where Φ(x) represents the Gaussian cumulative distribution function (under neutrality) and piHS is the two-sided *p*-value associated with the neutral hypothesis (i.e., no selection). The maximum allowed gap between two SNPs was set to 500 Kb, and 1-Mb sliding windows that partially overlapped 10 kb with adjacent windows were set. Candidate regions of positive selection were defined as genomic regions having an unusual clustering of SNPs with high iHS statistics (≥3). This was estimated as the proportion of SNPs surpassing the significance threshold of log10 (*p* value) = 3, equivalent to a *p*-value of 0.001. Candidate regions of positive selection were identified as windows with the top 1% density of high iHS SNPs.

#### 2.8.2. Cross-Population Extended Haplotype Homozygosity (XP-EHH)

A pairwise comparison was performed for Bartlow Combine and Kokstad Nguni cattle populations to identify genomic regions under increasing differentiation using XP-EHH (cross-population extended haplotype homozygosity). To detect alleles with higher frequency to the point of fixation or near-fixation in the Bartlow Combine population compared to the Kokstad Nguni cattle population, the XP-EHH scores were computed using the rehh R package v2.0.4 with default parameters [42]. FastPhase software [42] was used to phase the haplotypes, and XP-EHH scores were calculated for each haplotype within a population. Since XP-EHH searches for unusually extended haplotypes, at least three consecutive SNPs are required to be over the threshold for this analysis to be considered conservative, the threshold was determined using the log (*p*-value).

### 2.9. Annotation of Signatures of Selection Genomic Regions

Genes overlapping the genomic region under selection were determined using the ARS-UCD1.2/bosTau9 bovine reference genome [44] and the intersectBed command of BEDTools [45]. Similar to Liu et al. [46], the potential selection regions were defined by extending 200 kb both upstream and downstream of the potential selection signatures. A Venn diagram was constructed by an online tool [36] to depict genes common or unique between the two populations.

Both the unique and common genes were functionally annotated by performing Kyoto Encyclopedia of Genes and Genomes (KEGG) pathway analysis [47] and Gene Ontology (GO) [48] enrichment analysis using the Database for Annotation, Visualization, and Integrated Discovery (DAVID v6.8) [49]. Significant GO terms provided insight into the functional characteristics of annotated genes. The analyses allowed the identification of molecular functions, biological processes, cellular components, and pathways for the genes included in regions under selection. In addition, the QTL regions that spanned the signatures of selection were detected by mapping selected regions under selection onto QTL sections using data from the Animal QTL database [50].

## 3. Results

### 3.1. SNP Quality Control, MAF and F_IS_ per Population

A total of 643,275, 650,317, and 650,430 SNPs remained after quality control for the Bartlow Combine, Kokstad, and overall populations, respectively, and were utilized for downstream analysis, as illustrated in Table 1.

**Figure 1:**
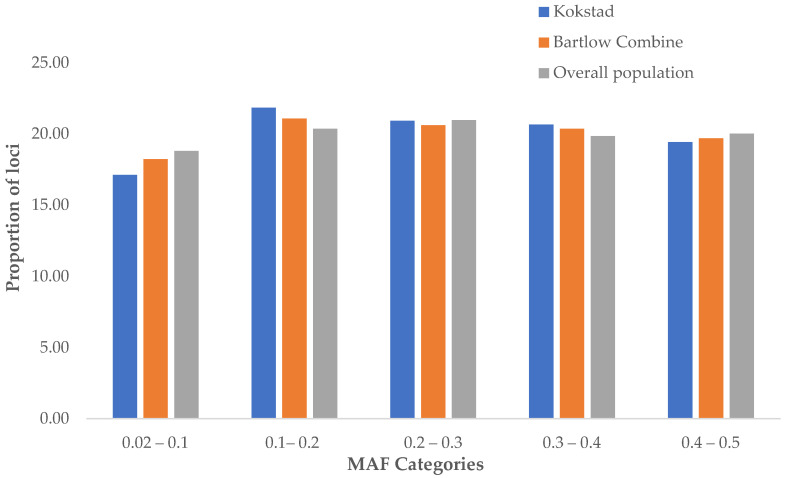
Distribution of minor allele frequencies in Bartlow Combine, Kokstad, and overall Nguni cattle populations. Proportion of the SNPs (Y-axis) from the HD chipset after quality control found inside each MAF category (X-axis) was averaged for both populations.

The distribution of MAF for each population is shown in Figure 1. About 45% of the SNPs had an MAF higher than 0.3 across herds. The frequency of SNPs in the different MAF categories were similar between the two herds.

### 3.2. PCA Genetic Clustering

**Figure 2:**
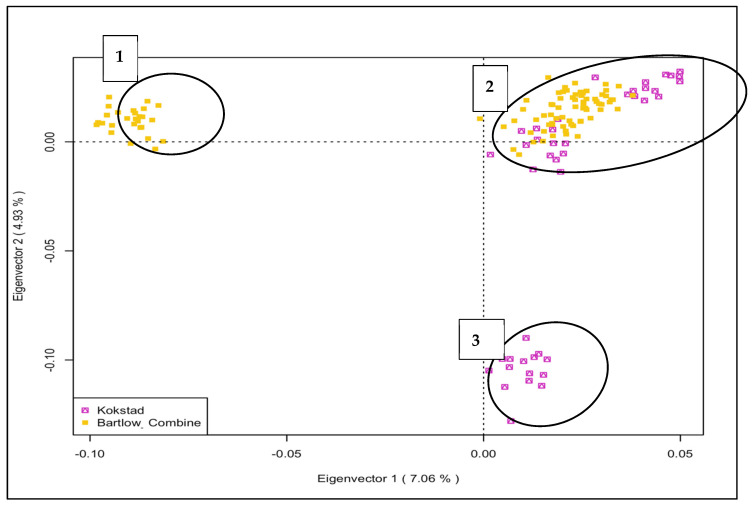
PCA−based clustering of Bartlow Combine and Kokstad Nguni cattle populations.

PC1 and PC2 explained 7.12% and 4.93% of the total genetic variation, respectively, and reported three genetic clusters (Figure 2 Genetic cluster 1 and 3 consisted of Bartlow Combine and Kokstad, respectively, while genetic cluster 2 had a mixture of both Bartlow Combine and Kokstad animals, suggestive of a common ancestral population between the two herds.

### 3.3. Linkage Disequilibrium Patterns and LD Decay

The average *r*^2^ ± SD between adjacent SNP across all chromosomes was 0.413 ± 0.219 for Bartlow Combine, 0.402 ± 0.209 for Kokstad, and 0.417 ± 0.222 for overall Nguni cattle populations. The LD chromosomal distribution is illustrated in Table 2.

The pattern of LD was significantly different among various chromosomes in each of the herds’ population (Table 2). A positive correlation in mean LD was observed between the Bartlow Combine and Kokstat herds (Figure S1: Supplementary Material File S1).

**Figure 3:**
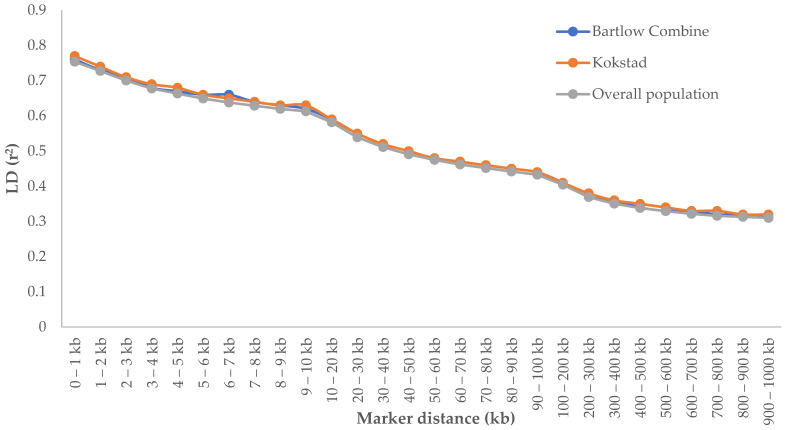
Linkage disequilibrium (*r^2^*) between adjacent SNPs pairs separated by different distances in Bartlow Combine, Kokstad, and overall Nguni cattle populations.

Genome-wide average LD (*r*^2^) decreased with increasing genetic distance between markers for all populations. The differences in *r*^2^ values observed in Bartlow Combine, Kokstad, and overall Nguni cattle populations, across all genetic distances, were very small, as illustrated in Figure 3. As expected, the maximum average *r*^2^ values for Bartlow Combine (0.76 ± 0.28), Kokstad (0.77 ± 0.27), and overall population (0.75 ± 0.28) were observed at a short distance (0–1 kb). Across populations, there was a lower LD that progressively declined with increasing genomic distance, especially for distances higher than 10 kb. The most rapid decline was observed over the first 100 kb. However, there were very small differences in LD decay between the Bartlow Combine, Kokstad, and overall populations.

### 3.4. Haplotype Frequencies and Haplotype Block Structure

A total number of 77,305, 66,237, and 84,182 haplotype blocks covering a total of 1570.09 Mb and 1367.42 Mb and 61.99%, 53.96%, and 65.05% of the genome were observed in the Bartlow Combine, Kokstad, and overall Nguni cattle populations, respectively (Table 3). The average length of the haplotype blocks was 20.31 kb, 20.64 kb, and 19.24 kb across chromosomes in the Bartlow Combine, Kokstad, and overall populations, respectively. The haplotype frequency was 0.23 in all Nguni populations.

**Figure 4:**
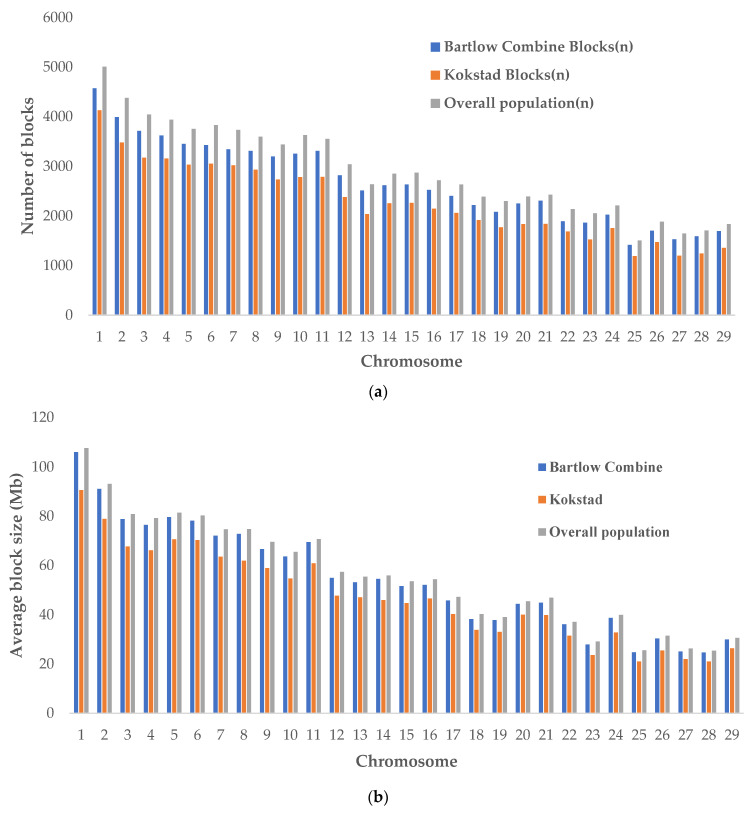
(**a**) Distribution of haploblocks per chromosome in Bartlow Combine, Kokstad, and overall Nguni cattle populations. (**b**) The average block size in the genomes of Bartlow Combine, Kokstad, and overall Nguni cattle populations.

The distribution of genome-wide haplotype blocks within the three populations is shown in Figure 4a. Large amounts of variation in haplotype block structure and the size between chromosomes were observed. Chromosome 1–11 are the largest in the cattle genome and were expected to have the largest and longer haplotype blocks. Chromosome 1 exhibited the most haplotype blocks at 4573 (31,571 SNPs), 4129 (28,136 SNPs), and 5008 (32,888 SNPs) in Bartlow Combine, Kokstad, and overall Nguni populations, respectively. The smallest number of haplotype blocks were identified on chromosomes 25 (1415 and 1192) in Bartlow Combine and Kokstad populations, respectively (Figure 4a, Table S2: Supplementary Material File S2 and Table S3: Supplementary Material File S3).

Chromosome 23 (53.16% and 45.09%) showed the smallest coverage, while Chr 1 (66.88%) and Chr 6 (59.63%) showed the greatest coverage in Kokstad and Bartlow Combine Nguni cattle populations, respectively Table S2: Supplementary Material File S2 and Table S3: Supplementary Material File S3). Chromosome 28 (55.42%) had the smallest coverage and Chr 2 exhibited the highest coverage in the overall population (Table S4: Supplementary Material File S4). The summary of the SNPs’ distribution and proportion involved in the haplotype block formation per chromosome for both Bartlow Combine and Kokstad Conservation herds is also presented in Table S2: Supplementary Material File S2 and Table S3: Supplementary Material File S3. In this analysis, we observed a small proportion of haplotype blocks containing more than 10 SNPs in each population. Overall, 74.88% (485,025) of all SNPs in the Bartlow Combine population and 65.44% (429,462) of all SNPs in the Kokstad population were clustered into haplotype blocks. The average block size in the overall population was higher than that of the Bartlow Combine and Kokstad populations in all autosomes (Figure 4b).

**Figure 5:**
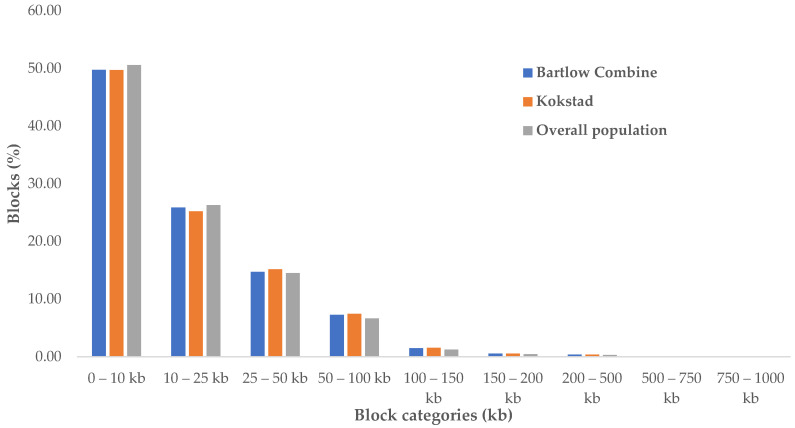
Proportion of haploblocks per block length category in Bartlow Combine, Kokstad, and overall Nguni cattle populations.

**Figure 6:**
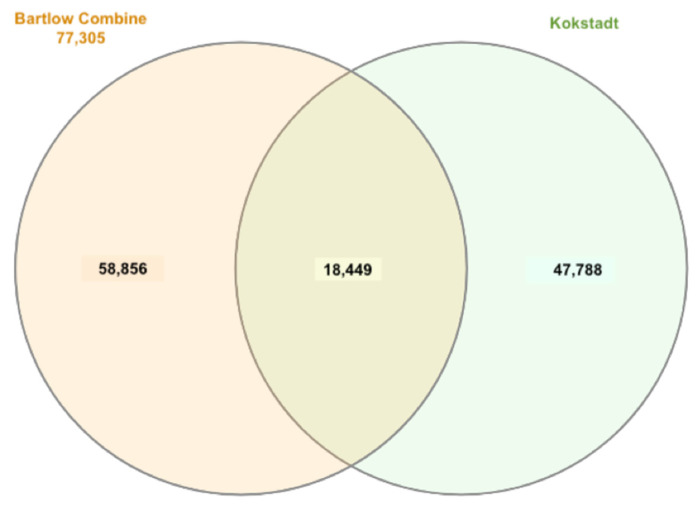
Venn diagram of shared and unique haplotype blocks between Bartlow Combine and Kokstad Nguni populations.

The number of blocks that were above 500 kb was almost the same in all three populations (Table S5: Supplementary Material File S5). About 50% of the identified haploblocks were located in the 0–10 kb length category in all populations (Figure 5). We compared the shared and unique haplotype block regions on chromosomes across populations. A total of 18,449 haploblocks were shared between the two populations, and 58,856 and 47,788 haploblocks were unique to Bartlow Combine and Kokstad populations, respectively (Figure 6).

### 3.5. Effective Population Size

**Figure 7:**
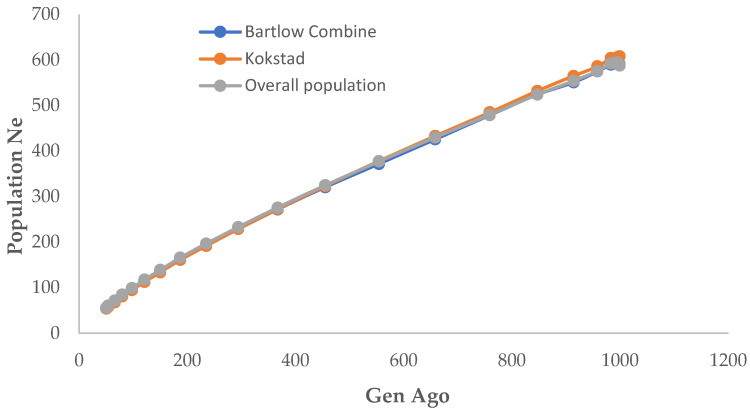
Estimated N_e_ for Bartlow Combine and Kokstad population over time based on linkage disequilibrium data.

The genome-wide estimate of N_e_ was computed based on linkage disequilibrium between SNPs, with sample size, mutation, and recombination rate being taken into account. Each line depicts the trend in effective population size across generations. The result showed that all populations had experienced a rapid decline, including the most recent generation (Figure 7). N_e_, 294 generations ago, was approximately 229 in Bartlow Combine and Kokstad herds, and was 233 in the overall population (Table S6: Supplementary Material File S6.

In the fifth generation (from the present), N_e_ was 55–98, 54–95, and 56–99 in Bartlow Combine, Kokstad, and overall population, respectively. Overall, N_e_ decreased from ~590, 608, and 588 (999th generation ago) to 55, 54, and 56 (50th generation ago) in Bartlow Combine, Kokstad, and overall populations, respectively. The estimated N_e_ in the three populations revealed that the herd formation was the same within the last 100 generations.

### 3.6. Selective Sweeps in Bartlow Combine and Kokstad Nguni Cattle Populations

#### 3.6.1. Recent Positive Selection Identified by iHS

**Figure 8:**
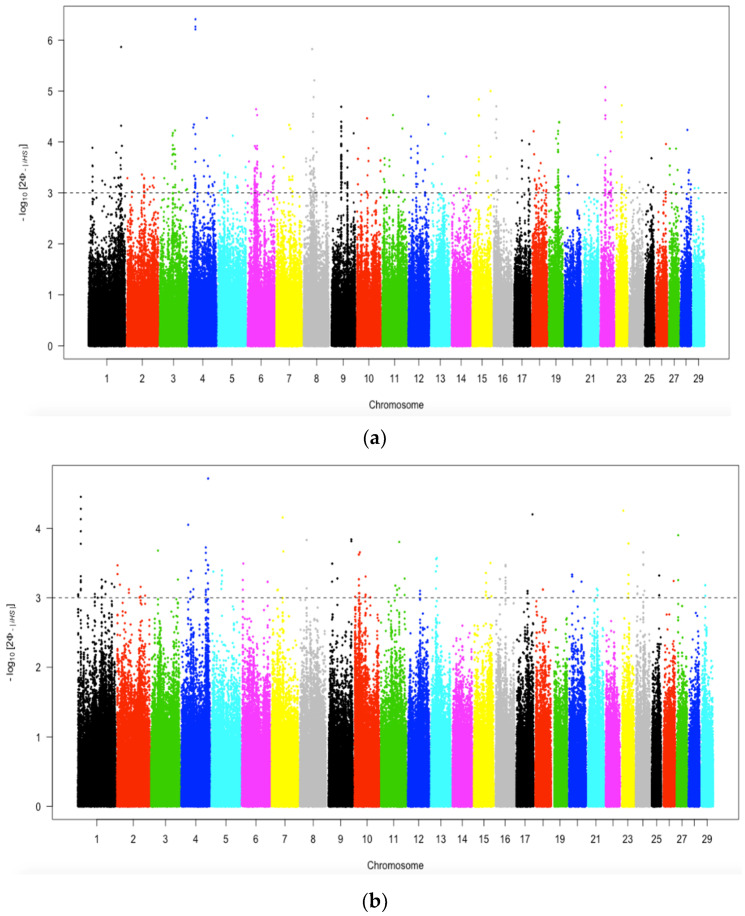
Genome−wide distribution of selection signatures detected by iHS on 29 autosomes in the Bartlow Combine (**a**) and Kokstad (**b**) Nguni cattle populations. The dotted horizontal line shows the cut-off |iHS| value to call SNP outliers.

Figure 8a,b illustrates the genomic regions under selection within the Bartlow Combine and Kokstad herds, respectively. A total of 553 and 166 SNPs with top 1% normalized iHS values were considered to be the candidate regions for selection in the Bartlow Combine and Kokstad populations, respectively (Table 4; Table S7: Supplementary Material File S7). Plots showing both positive and negative signatures of selection in Bartlow Combine and Kokstad populations are presented in Figure S8: Supplementary Material File S8.

The most extreme iHS peaks were on BTA 1, 4, and 8 for the Bartlow Combine Nguni cattle population and chromosomes 1, 4, 8, 17, and 23 for the Kokstad population. The highest |iHS| value were 6.41 (SNP: BovineHD0400008236) and 4.72 (SNP: BovineHD0400032209) for Bartlow Combine and Kokstad populations, respectively. The results for the overall population are presented in Figure S8: Supplementary Material File S8.

#### 3.6.2. Positive Selection Identified by Cross-Population Extended Haplotype Homozygosity

**Figure 9:**
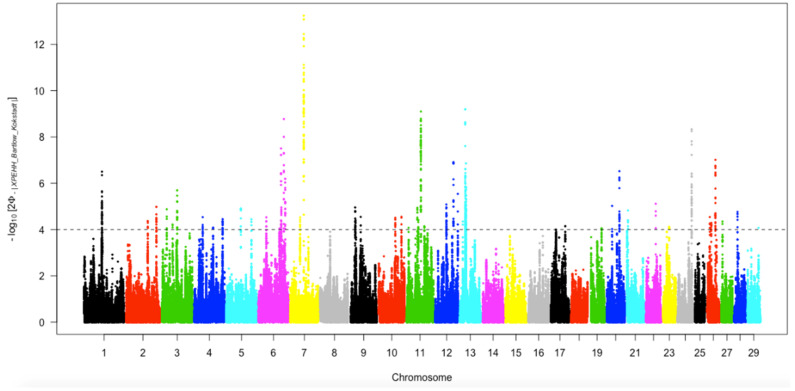
Genome−wide distribution of selection signatures detected by XPEHH across all autosomes in Bartlow Combine and Kokstad Nguni cattle populations.

Figure 9 depicts the genome-wide distribution of the outliers on each autosome that were detected separately by XP-EHH in Bartlow Combine and Kokstad Nguni cattle populations. The significance cut-off values (4.0) were assigned from the distribution of standard normalizing the XP-EHH. Moreover, 57 SNPs with values above the cut-off threshold were considered candidates of selection signatures (Table 4). The selection signatures were not uniformly distributed across the whole genome in the two populations.

#### 3.6.3. Genomic Annotation

The longest haplotype blocks were observed at BTA7 in both populations, i.e., Bartlow Combine (999.05 kb, 185 SNPs, location: 51661856 bp—52660903 bp) and Kokstad (999.37 kb, 193 SNPs, location: 51579684 bp—52579053 bp) populations. The two blocks identified in Bartlow Combine and Kokstad populations overlaps the genes such as *CD14*, *WDR55*, *PCDHA3*, *RF00026*, etc., (Table S9: Supplementary Material File S9). A total of 7570, 1677, and 94 genes were identified in the 553, 166, and 505 significant iHS genomic regions in the Bartlow Combine, Kokstad, and overall populations, respectively (Table 4). A total of 2423 genes corresponding to 2208 genes with known IDs that overlapped 57 selection signatures detected by XP-EHH contrasting the Bartlow Combine from the Kokstad animals (Table 4).

**Figure 10:**
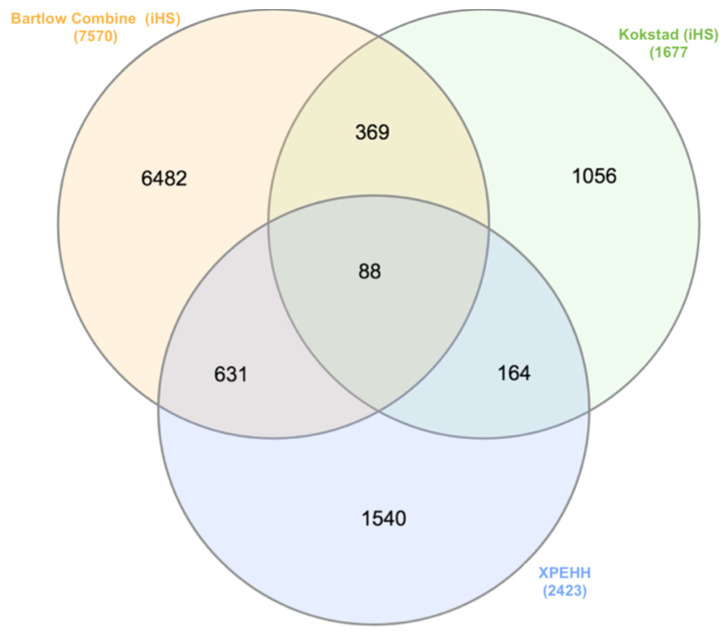
Venn diagram comparing the unique and shared genes under selection between the Bartlow Combine and Kokstad populations.

Shared and unique genes within/between the two populations and signatures of selection methods are represented in Figure 10. A total of 369 genes were shared between the two populations, and 6482 and 1056 genes were unique to the Bartlow Combine and Kokstad populations, respectively. A total of 631 genes were shared between the Bartlow Combine (iHS) and XP-EHH test genes, and 164 genes were shared between the Kokstad (iHS) and XP-EHH test genes. Eighty-eight genes were common in Bartlow Combine (iHS), Kokstad (iHS), and XP-EHH test genes, while 1540 genes were only detected when using the XP-EHH method.

DAVID, GO terms, and QTL analysis was performed for genes (i) unique to Kokstat, (ii) unique to Bartlow Combine, (iii) common between the two populations, and (iv) on those from XP-EHH. The candidate genes that were detected in the Bartlow Combine population encompassed a wide spectrum of molecular functions, biological processes, cellular components, and pathways, and were enriched in 183 gene ontology (GO) and 61 KEGG pathways, as shown in Table 4. These included GO terms for negative regulation of fat cell differentiation, T cell activation involved in immune response, defense response to protozoan, humoral immune response, negative regulation of inflammatory response, amongst other terms (Table S10: Supplementary Material File S10). Some of the KEGG pathways included those involved in *Staphylococcus aureus* infection, lipid and atherosclerosis, vascular smooth muscle contraction, *Salmonella* infection, and melanogenesis. The selected regions overlapped with QTLs associated with productive, functional (including resistance to diseases), and morphological traits (Table S11: Supplementary Material File S11).

The 1056 genes unique to the Kokstad cattle were linked to GO terms involved in the positive regulation of cytokine production involved in the inflammatory response, antigen processing and presentation, cellular response to cAMP, the canonical *Wnt* signaling pathway, and the defense response to Gram-negative bacterium (Table S12: Supplementary Material File S12). Some of the KEGG pathways identified included that for salivary secretion, melanogenesis, and salivary secretion. The overlapping QTL records were associated with body weight, milk fat, calving ease, milk production, milk protein, body weight at birth, antibody-mediated immune response, and fat thickness at the 12th rib (Table S13: Supplementary Material File S13). The annotation of genes identified in the overall population revealed 23 GO terms which were mainly involved in cardiac muscle hypertrophy, mammary gland epithelium development, regulation to epidermal growth factor receptor signaling pathways.

The annotation of genes common (identified by iHS) in both Bartlow Combine and Kokstad Nguni populations revealed 49 GO terms and 2 KEGG pathways. The identified GO terms were involved in processes such as the maintenance of gastrointestinal epithelium, the defense response to Gram-negative bacterium, and antigen processing and presentation (Table S14: Supplementary Material File S14).

The annotation of genes identified by the XP-EHH revealed 137 GO terms and 37 KEGG pathways (Table S15: Supplementary Material File S15). This set of genes encompassed a wide spectrum of molecular functions, biological processes, cellular components, and KEGG pathways and included antigen processing and the presentation of peptide or polysaccharide antigen via *MHC* class II, the defense response to bacterium, an inflammatory response, the detection of chemical stimulus involved in the sensory perception of bitter taste, the epoxygenase P450 pathway, antimicrobial humoral immune response mediated by antimicrobial peptide, an immune response, defense response, and adaptive immune response, the regulation of MAPK cascade, the activation of MAPK activity, and DNA methylation GO terms.

A total of 907 bovine QTLs overlaps (Table S16: Supplementary Material File S16) were observed, which were associated with traits of economic importance, such as *MTX2*, *U6*, *THSD7A*, *TMEM106B*, *DTNB*, *DNMT3A*, *bta-mir-1301*, *POMC*, *EFR3B*, *DNAJC2*, *ZNF280A*, *YWHAH*, *SLC5A1*, *SLC5A4*, *ZNF280B*, and *ADCY3* for carcass and body weight, ABCA12, *SNORA70*, *ATIC*, *FN1*, and *bta-mir-2285l* for reproduction, *ZNF830*, *CCT6B*, *SNORA70*, *THSD7A*, *TMEM106B*, and *TMEM132E* for residual feed intake, and *RASL11B*, *SCFD2*, *GPAT4*, *bta*-*mir*-*486*, 5S_*rRNA*, *GOLGA7*, *SFRP1*, *NKX6*-3, and *GINS4* for milk production.

## 4. Discussion

Conservation of animal genetic resources focuses not only on endangered breeds but also on those that are underutilized. Locally adapted breeds are constantly in danger of becoming extinct, especially when local populations favor imported breeds. The Bartlow Combine and Kokstad Nguni cattle conservation herds were established in an attempt to conserve the eroding Nguni genetic resources in South Africa. The two herds and other conservation and research population are often used for research purposes as representatives of the Nguni breed. As in any other species [13], conservation herds are at a risk of losing their original genetic diversity and of diverging from founding breeds over time. Whilst continuous monitoring of the diversity and evolution of the conservation herds is required, this is seldom done because of limited resources. Ideally, the genetic architecture of conservation herds should be measured initially and routinely when the herds are established to monitor changes.

This study characterized the pattern of LD, haplotype block structures, effective population sizes, and the genomic signatures of selection in the Bartlow Combine and Kokstad Nguni cattle conservation herds. As both were established from the same breed, the study hypothesized that the two herds will be genetically similar and present the same genomic architectures. Differences between the two herds would imply the presence of unique genetic diversity or a possibility of different forces of evolution acting upon these two conservation herds.

Minor allele frequency (MAF) is commonly utilized in population genetic studies because it allows researchers to distinguish between common and uncommon variations. The MAF values were similar between the Bartlow Combine cattle, Kokstad Nguni cattle, and the overall population (Table 1). The MAF means across all autosomes reported in the present study, which were (0.25 ± 0.14) for Bartlow Combine, (0.26 ± 0.14) in the Kokstad cattle, and (0.25 ± 0.14) in the overall population, were comparable to those reported by Makina et al. [3] for Nguni cattle (0.26 ± 0.13) and other South African indigenous breeds of Afrikaner (0.25 ± 0.13), Drakensberger (0.27 ± 0.13), and Bonsmara (0.26 ± 0.13).

The Nguni MAF results from the study of Zwane et al. [51] also reported similar MAF in Nguni cattle (0.27 ± 0.133) to those observed in the present study for the Bartlow Combine and Kokstad conservation herds. The observed MAF results for both Bartlow and Kokstad indicate that the two research populations are (i) not at risk of extinction and (ii) are under low and insignificant selection since the values are comparable to those of field populations including that of Nguni field populations reported by Makina et al. [3] and Zwane et al. [51].

Natural selection, genetic drift, and gene flow affect allele frequencies [52], and these mechanisms do not appear to be in play in the Nguni herds used in this study. This implies that the conservation herds have not evolved significantly from the original or field populations. Furthermore, results demonstrated genetic similarities between the Bartlow Combine and Kokstad cattle that presented similar MAF profiles. This observation is an indication that the two herds are (i) genetically similar, having been found from the same breed, and (ii) are subjected to similar evolutionary processes, resulting in them maintaining a similar MAF even with the field Nguni cattle breeds.

The occurrence of the highest number of SNPs in the MAF category 0.1–0.2 is a common observation in indicine breeds, and opposite tendency is observed in the taurine breeds, where most of the SNPs are located in the last two categories [53,54]. Similar results were reported by O’Brien et al. [55], who studied LD levels in *Bos indicus* and *Bos taurus* cattle using medium- and high-density SNP chips. Karimi et al. [56], however, reported that composite and taurine cattle breeds had a greater number of SNPs in the 0.3–0.4 and 0.4–0.5 MAF categories compared to the indicine breed. Both the Bartlow Combine and Kokstad cattle therefore behaved in a similar pattern as indigenous and purebred breeds or populations.

LD is a fundamental approach for identifying the genetic structure of economically important traits in livestock species [57]. Karimi et al. [56] stated that the average extent of LD is highly variable in different studies depending on the study population and the threshold used to measure LD. In the present study, the average LD between adjacent SNP values across all chromosomes was 0.413 ± 0.219 for Bartlow Combine Nguni cattle, 0.402 ± 0.209 for Kokstad cattle, and (0.417 ± 0.222) for the overall Nguni cattle population (Table 2). Makina et al. [3] reported LD estimates of 0.47, 0.37, 0.37, 0.37, 0.46, and 0.45 for Afrikaner, Nguni, Drakensberger, Bonsmara, Angus, and Holstein, respectively.

LD estimates obtained in the Bartlow Combine and Kokstad research stations are therefore similar though slightly higher than those reported by Makina et al. [3], who studied field populations. The results from this study imply that the two Nguni conservation populations have maintained their genetic diversity with minimum effects of selection and other evolutionary forces. When the two research stations were formed, different Nguni ecotypes were bought from villages [58], and that might be the reason for the observed small differences in LD estimates between the Nguni field [3] and the Bartlow Combine and Kokstad populations used in this study. The relatively high LD observed in both herds is expected and, according to Rogers [59], likely related to a higher ancestral relatedness and to a historically smaller N_e_ in local breeds and established herds.

A small finite population size is normally reflected by high levels of linkage disequilibrium [19]. Selection plays a significant role in the extent of LD; however, its influence is associated with specific genes [60]. There was a high level of relatedness between the Bartlow Combine and the Kokstad conservation populations. The two studied conservation flocks, Bartlow Combine and Kokstad herds, were established using a small number of breeding animals and are affected by strong genetic drift, which may explain the slightly high LD estimates observed in the two populations.

The LD decreased with increasing physical distance between markers in both Nguni herds, as well as in the overall population (Figure 3). Similar results were reported by Makina et al. [3]. However, in the Nguni field population studied by [3], LD decayed more rapidly compared to the Bartlow Combine and Kokstad Nguni cattle conservation herds. In addition to the decrease in LD levels with the increased marker distance, the LD also showed variability among chromosomes and chromosomal regions, which may be a result of QTLs that have been under selection in different chromosomes and chromosomal regions. Many different factors, such as differences in the recombination rates between and within chromosomes, heterozygosity, selection effects, and genetic drift, might explain the variations in LD decay between the present study and previous studies. Karimi et al. [56] reported that the diversity in LD patterns observed in individual autosomes among populations could be a result of uneven selection pressures on QTLs distributed throughout the genome. Under this presumption, chromosomes harbouring quantitative trait loci (QTL) undergoing selection are expected to have higher LD compared to other chromosomes.

In our study, the highest LD values were observed on chromosome 7 in the Bartlow Combine population and in chromosomes 7 and 8 in the Kokstad population, which might imply the presence of QTLs with large effects that have been subjected to intense selection and generate high LD with neighbouring markers in the associated chromosomes. Chromosomes 7 and 8 harbour QTLs associated with milk production, meat traits, and disease or nematode resistance [61,62,63], which are traits important to the Nguni cattle that are used for both beef and milk production [1] and are reported to be robust to diseases and parasites [64].

Our study went on to examine haplotype diversity as a measure of genetic diversity in the Bartlow Combine and Kokstad cattle and observed similar haplotype frequency (0.23) in both herds. The slightly higher number of haplotypes and haplotype blocks in Bartlow Combine than the Kokstad population could be an effect of the differences in sample sizes [65]. Haploblocks reported in this study were larger than those reported by Wang et al. [66], who studied the prevalence of haplock structures in South African Nguni cattle using Illumina BovineSNP50 Beadchip data and found a total of 541 haploblocks covering 41.60 Mb of the cattle genome (UMD 3.0 bovine reference genome).

In the present study, the largest haploblocks were found on chromosome 1 (4573, 4129) in Bartlow Combine and Kokstad populations, respectively. The longest haplotype blocks were found in chromosome 7 (999.05 kb, 999.37 kb) in the Bartlow Combine and Kokstad populations, respectively. Wang et al. [66] also found the largest haploblocks on chromosome 1; however, the longest haplotype block was found on chromosome 10 (123 kb), and this haploblock was significantly smaller than the ones found in the Bartlow Combine and Kokstad populations.

The length of haplotype blocks in the Bartlow Combine and Kokstad populations were longer than those of the Nguni populations studied by Wang et al. [66] and Makina et al. [3]. This result is probably due to the Bartlow Combine and Kokstad research populations having been strongly selected in recent decades compared to the field Nguni populations in other studies. It has been established that the average LD decay with the increasing physical genomic distance between loci is more emphasized in crossbred and admixed populations than in purebred and highly selected populations [65,67].

The generational transfer of genetic resources between breeds is possible thanks to haplotype sharing [68]. The shared and unique haplotype blocks observed in this study revealed both similarities of the Bartlow Combine and Kokstad cattle as well as some level of the uniqueness of each conservation herd. The shared haplotype blocks observed in animals originating from the same breed could indicate the existence of conserved genomic regions. Population-specific haplotype blocks in these two herds, on the other hand, could be considered a valuable tool for identifying and protecting its genetic diversity, as they potentially indicate a genomic source of unique phenotypic features in each herd. According to Clark et al. [69] and Templeton et al. [70], the clustering of blocks is an indication of local hotspots of recombination, and our haplotype analysis clearly indicates that both the Bartlow Combine and Kokstad conservation populations have high haplotype diversity hotspots.

The effective population sizes obtained for both the Bartlow Combine and Kokstad Nguni cattle populations, as well as for the overall population, in this study, were in agreement with those reported by de Roos et al. [71]. The decrease in N_e_ observed in the two conservation herds might be related to an increase in inbreeding levels and a reduction in genetic variety, both of which are common in animals with small, finite populations [72]. When the two conservation herds were established, different Nguni ecotypes were bought from nearby villages, and it is possible that a small number of animals were used to establish the two populations. Makina et al. [3] observed a significantly higher N_e_ (~2500) in a Nguni field cattle population compared to the present study. Continuous genetic erosion and decline in these populations increases the risk of losing some economically important traits of the Nguni cattle breed. Effective population sizes were the same in both populations: 55 in the Bartlow Combine population and 54 in the Kokstad population. The Ne observed in both populations corresponded to similar *r*^2^ at 1000 kb (0.31 and 0.32) in Bartlow Combine and Kokstad populations, respectively. Overall, the LD, LD Decay, Haplotype, and N_e_ results are suggestive of the fact that the two conservation herds are genetically similar with minimum divergence between them and from the field populations analysed in previous studies. The results also demonstrated the impact of movement into conservation herds, which resulted in slightly lower effective population sizes and higher LD and haplotype diversity than would be observed in field populations.

The next set of analysis investigated signatures of selection in the genomes of the two conservation herds. Based on the PCA results, genetic cluster 2, which consisted of animals from both populations, was excluded from this analysis. Both the iHS and XP-EHH plots indicated that common selective pressure on both populations, probably due to a common founding breed and similar production goals, is contrasted with selection toward different genomic regions, most likely due to differences in habitats, genetic drift, effective population size, inbreeding, and recombination occurring between the two herds.

The melanogenesis pathway was enriched in both Bartlow Combine and Kokstad populations and involved genes such as *WNT3, RAB5A, ASIP, WINT9A, WLS, WNT9B, WNT11, MAPK1, WWTR1, G3BP, PIK3R3, FSHB, EDNRA, ADCY4*, and *ADCYAP1R1*. The presence of coat colour-related genes in both populations is in line with the common perception that coat colour is a breed-defining trait under selection in Nguni cattle [3]. Nine genes were involved the in cyclic adenosine monophosphate (cAMP)-signaling pathway, and these genes include *HCAR1, GNAI1, PIK3R3, FSHB, EDNRA, ADCY4, ADCYAP1R1,* and *HTR1E*. According to Bang and Zippin [73], cAMP is a second messenger that regulates numerous functions in both benign melanocytes and melanoma cells. In animals, an alpha-melanocyte-stimulating hormone (alpha-MSH) and an adrenocorticotropic hormone (ACTH) are primarily responsible for pigmentation [74]. Significant pathways such as MAPK signaling and oxidative stress response pathways were identified in both herds.

Other genomic regions under selection in both populations included those responsible for immune response mechanisms. Nguni cattle are known to be tolerant to different endemic parasitic diseases [5], therefore the immunity-related genes within the candidate regions identified in Bartlow Combine and Kokstad populations (*BOLA-N* (Class IB *MHC* Antigen QA-2-Related), *BOLA* (Class IB *MHC* Antigen QA-2-Related), and *Rab-8B*) are potential targets of natural selection. Exon 2 of the *BoLA*-*DRB3* gene is extremely important and is involved in the T-cell response to pathogens [75]. The *MHC* is a genetically diverse region in natural populations that is involved in the production of glycoproteins that adhere to foreign substances and redirect them to important immune system components [76]. Marufu et al. [14] conducted a study to determine the prevalence and loads of gastrointestinal parasites in Nguni and non-descript cattle on semi-arid rangelands and observed that the Nguni cattle had lower (*p < 0.05*) mean egg counts (MEC) for *S. papillosus* than local crossbreds.

Another enriched Go-term was the maintenance of gastrointestinal epithelium, which is defined as the protection of epithelial surfaces of the gastrointestinal tract from proteolytic and caustic digestive agents. Cattle are natural reservoirs for a range of important enteric pathogens, including *Salmonella*, *Escherichia coli*, *Mycobacterium avium* subspecies *paratuberculosis* and *Cryptosporidium parvum*, and it is crucial to understand how these pathogens interact with the bovine intestinal epithelium [77]. Two KEGG pathways, bta00860: Porphyrin metabolism and bta04970: Salivary secretion, were identified in both herds. According to Beauchemin et al. [78], salivary secretion elevates rumen pH, which improves digestion. The genes involved in processes such as immunity lie within the selection signatures that were identified in both Bartlow Combine and Kokstad conservation herds. These results may suggest that both herds are predisposed to diseases and parasites, and animals in these research stations have developed natural resistance against such.

Furthermore, a number of QTLs relating to milk production, meat, and carcass traits and pigmentation overlapped with genes in region under selection in both populations. These results were expected because the Nguni is being selected for milk production, meat production, coat colour, as well as tick or disease resistance. The presence of these GO-terms only in genes unique to either the Bartlow or Kokstad population might be suggestive of different natural and artificial selection pressures between the two herds. Such differences could be due to nutrition, management, and environmental and climatic factors [79].

## 5. Conclusions

This study provided a comprehensive analysis of the genomic architecture of Nguni cattle conservation herds using the Illumina^®^ BovineHD Genotyping BeadChip. Overall, similarities were observed between the two herds and also with the field populations based on results from previous studies. The slightly high levels of LD at short distances reported in this study might be due to the fact that Bartlow Combine and Kokstad are conservation research herds with smaller and more finite population sizes, whilst the other studies characterized field populations. The study provided evidence of a rapid decline in the effective population size in both Bartlow Combine and Kokstad conservation herds.

Several candidate genomic regions showing a positive selection signature were identified using two haplotype-based methods. Our analyses revealed important genes related to coat colour, adaptation, immune response, and production traits in both studied research populations. The use of two different statistical approaches (iHS and XP-EHH) facilitated the wider spectrum of the detection of selection signatures within and between Bartlow Combine and Kokstad Nguni cattle populations. The findings can provide valuable knowledge for further functional genomic studies, GWAS, and genomic selection, implementing breeding schemes and conservation programs.

## Figures and Tables

**Table 1:** Number of autosomal SNPs and individuals before (pre-) and after (post-) quality control (QC) per population.

Population	# Animals Pre-QC	# SNPs Pre-QC	# SNPs Post-QC	#Animals Post-QC	Genotyping Rate	Mean IBD	*F_IS_*	Mean MAF
Bartlow Combine	99	777,962	643,275	85	0.998	0.041	−0.014	0.25 ± 0.14
Kokstad	44	777,962	650,317	42	0.999	0.069	−0.028	0.26 ± 0.14
Overall	143	777,962	650,430	127	0.998	0.032	−0.011	0.25 ± 0.14

# = Number of; *F_IS_* = Inbreeding Coefficient; IBD = Identity By Descent; MAF = Minor allele frequency.

**Table 2:** Summary of mean, median, and standard deviation of *r*^2^ values along Bartlow Combine Kokstad, and overall Nguni cattle chromosomes.

	Bartlow Combine		Kokstad		Overall Population	
Chr	Mean LD ± SD (*r^2^*)	Median (*r^2^*)	Mean LD ± SD (*r^2^*)	Median (*r^2^*)	Mean LD ± SD (*r^2^*)	Median (*r^2^*)
1	0.419 ± 0.221	0.337	0.413 ± 0.214	0.335	0.422 ± 0.223	0.338
2	0.423 ± 0.225	0.338	0.412 ± 0.215	0.335	0.422 ± 0.225	0.338
3	0.418 ± 0.223	0.332	0.401 ± 0.209	0.324	0.420 ± 0.224	0.334
4	0.410 ± 0.213	0.332	0.408 ± 0.206	0.337	0.412 ± 0.213	0.335
5	0.419 ± 0.222	0.336	0.411 ± 0.213	0.334	0.421 ± 0.222	0.339
6	0.418 ± 0.220	0.338	0.413 ± 0.214	0.335	0.421 ± 0.223	0.337
7	0.436 ± 0.232	0.347	0.417 ± 0.216	0.339	0.433 ± 0.228	0.348
8	0.429 ± 0.221	0.350	0.417 ± 0.215	0.340	0.428 ± 0.221	0.349
9	0.417 ± 0.224	0.331	0.409 ± 0.214	0.330	0.421 ± 0.225	0.334
10	0.410 ± 0.217	0.328	0.398 ± 0.206	0.323	0.412 ± 0.219	0.330
11	0.419 ± 0.225	0.332	0.408 ± 0.213	0.331	0.422 ± 0.227	0.335
12	0.420 ± 0.220	0.338	0.402 ± 0.210	0.324	0.424 ± 0.223	0.341
13	0.414 ± 0.216	0.335	0.406 ± 0.207	0.332	0.418 ± 0.216	0.338
14	0.407 ± 0.211	0.330	0.405 ± 0.208	0.330	0.409 ± 0.212	0.330
15	0.415 ± 0.217	0.334	0.407 ± 0.214	0.329	0.417 ± 0.220	0.335
16	0.416 ± 0.224	0.330	0.406 ± 0.216	0.325	0.423 ± 0.227	0.339
17	0.410 ± 0.221	0.325	0.400 ± 0.210	0.322	0.417 ± 0.225	0.330
18	0.407 ± 0.221	0.319	0.387 ± 0.198	0.314	0.413 ± 0.223	0.326
19	0.408 ± 0.215	0.327	0.397 ± 0.204	0.322	0.415 ± 0.218	0.334
20	0.409 ± 0.217	0.327	0.409 ± 0.213	0.330	0.417 ± 0.222	0.332
21	0.424 ± 0.224	0.341	0.410 ± 0.216	0.328	0.428 ± 0.227	0.344
22	0.422 ± 0.225	0.337	0.400 ± 0.208	0.323	0.421 ± 0.225	0.334
23	0.399 ± 0.217	0.314	0.384 ± 0.203	0.307	0.409 ± 0.222	0.320
24	0.405 ± 0.214	0.325	0.396 ± 0.205	0.321	0.410 ± 0.218	0.327
25	0.410 ± 0.223	0.322	0.387 ± 0.207	0.378	0.421 ± 0.230	0.330
26	0.390 ± 0.208	0.310	0.379 ± 0.198	0.306	0.399 ± 0.215	0.314
27	0.406 ± 0.220	0.321	0.399 ± 0.207	0.324	0.416 ± 0.225	0.329
28	0.398 ± 0.211	0.318	0.385 ± 0.203	0.310	0.406 ± 0.216	0.323
29	0.401 ± 0.212	0.320	0.391 ± 0.201	0.316	0.406 ± 0.215	0.324
**Mean**	**0.413 ± 0.219**	**0.330**	**0.402 ± 0.209**	**0.328**	**0.417 ± 0.222**	**0.333**

*r***^2^**: Linkage disequilibrium; LD: Linkage disequilibrium; SD: Standard deviation.

**Table 3:** Summary statistics for haploblocks across Bartlow Combine, Kokstad, and overall Nguni cattle populations.

Populations	Bartlow Combine	Kokstad	Overall Population
Blocks (*n*)	77,305	66,237	84,182
Total block length^a^ (Mb)	1570.09	1367.42	1619.33
% Coverage	61.99	53.96	65.05
Mean block length (kb)	20.31	20.64	19.24
Haplotype frequency	0.23	0.23	0.23
Median block length (kb)	10.08	10.10	9.82
Max block length (kb)	999.05	999.37	999.05
No. of SNPs in blocks	485,025	429,462	508,313
SNP % in blocks	74.88	65.44	78.27
Mean num of SNPs in blocks	6.27	6.48	6.04
Max num of SNPs in blocks	185.00	193.00	186.00

**Table 4:** Regions under positive selection in both populations.

Populations	SNPs under Selection	Genes	GO-Terms	KEGG Pathways
**iHS test**				
Bartlow Combine	553	7570	183	61
Kokstad	166	1677	68	11
Overall population	505	94	23	0
**XPEHH**				
Bartlow Combine vs Kokstad	57	2423	137	37

## Data Availability

The SNP genotypes of the Nguni cattle generated for this project are available on https://osf.io/t36bw/?view_only=2a7687c679a042abbf5c5449ab926d43 (accessed on 8 July 2022) and can be downloaded upon request.

## Supplementary Materials

The following supporting information can be downloaded at: https://www.mdpi.com/article/10.3390/ani12162133/s1, Figure S1: Supplementary Material File S1; Table S2: Supplementary Material File S2; Table S3: Supplementary Material File S3; Table S4: Supplementary Material File S4; Table S5 Supplementary Material File S5; Table S6: Supplementary Material File S6; Table S7: Supplementary Material File S7; Figure S8: Supplementary Material File S8; Table S9: Supplementary Material File S9; Table S10 Supplementary Material File S10; Table S11 Supplementary Material File S11; Table S12 Supplementary Material File S12; Table S13 Supplementary Material File S13; Table S14 Supplementary Material File S14; Table S15 Supplementary Material File S15; Table S16 Supplementary Material File S16.
