# Supplementary material for: Linkage Disequilibrium, Haplotype Block Structures, Effective Population Size and Genome-Wide Signatures of Selection of Two Conservation Herds of the South African Nguni Cattle
Source: Animals (Basel). 2022 Aug 19;12(16):2133. doi: 10.3390/ani12162133 (PMC9405234; doi:10.3390/ani12162133)
Supplement: Supplementary file 1 [file animals-12-02133-s001.zip › Table S2 - Additional file S2.pdf]

Table S2: Bartlow haplotype block summary per chromosome (chr)

Chr	Chr length (Mb)	SNP(n) per Chr	Mean Block length (Mb)	Blocks (n)	Block coverage length (Mb)	% Chr block coverage	SNPs (n) in blocks	% SNPs in blocks	SNP average per Chr
1	158.53	40554	0.02±0.03	4573	106.03	66.88	31571	77.85	6.90
2	136.23	34913	0.02±0.03	3991	91.04	66.83	27372	78.40	6.86
3	121.01	31399	0.02±0.03	3717	78.84	65.15	23852	75.96	6.42
4	120	30577	0.02±0.03	3623	76.48	63.73	22883	74.84	6.32
5	120.09	30163	0.02±0.04	3455	79.6	66.28	23146	76.74	6.70
6	117.81	30574	0.02±0.04	3428	78.18	66.36	23500	76.86	6.86
7	110.68	29499	0.02±0.04	3343	72.07	65.12	21785	73.85	6.52
8	113.32	29207	0.02±0.03	3314	72.767	64.21	21758	74.50	6.57
9	105.45	27128	0.02±0.03	3200	66.66	63.21	20664	76.17	6.46
10	103.31	26339	0.02±0.03	3254	63.67	61.63	19831	75.29	6.09
11	106.98	27508	0.02±0.03	3311	69.51	64.97	21404	77.81	6.47
12	87.22	22987	0.02±0.03	2822	54.91	62.96	17542	76.31	6.22
13	83.47	20401	0.02±0.03	2512	53.14	63.66	15215	74.58	6.06
14	82.4	21844	0.02±0.03	2619	54.53	66.18	16651	76.23	6.36
15	85.01	21301	0.02±0.03	2635	51.68	60.79	15758	73.98	5.98
16	81.01	21416	0.02±0.03	2524	52.08	64.29	16469	76.90	6.53
17	73.17	19633	0.02±0.03	2404	45.84	62.65	14787	75.32	6.15
18	65.82	17294	0.02±0.03	2219	38.24	58.10	12815	74.10	5.76
19	63.45	16178	0.02±0.03	2082	37.81	59.59	11852	73.26	5.69
20	71.97	18744	0.02±0.03	2253	44.41	61.71	14030	74.85	6.23
21	69.86	19107	0.02±0.04	2309	44.88	64.24	14642	76.63	6.34
22	60.77	15604	0.02±0.03	1891	36.12	59.44	11360	72.80	6.01
23	52.5	13527	0.01±0.03	1863	27.91	53.16	9576	70.79	5.14
24	62.32	16753	0.02±0.03	2027	38.76	62.20	12654	75.53	6.24
25	42.35	11200	0.02±0.02	1415	24.77	58.49	8070	72.05	5.70
26	51.99	13383	0.02±0.03	1704	30.4	58.47	9735	72.74	5.71
27	45.61	11561	0.02±0.03	1530	25.08	54.99	8286	71.67	5.42
28	45.94	11643	0.02±0.02	1590	24.7	53.77	8406	72.20	5.29
29	51.1	12838	0.02±0.03	1697	29.98	58.67	9411	73.31	5.55
**Total**	**2489.37**	**643275**	**0.02±0.03**	**77305**	**1570.09**	**61.99**	**485025**	**74.88**	**6.27**

% Chr block coverage = Block coverage length (Mb)/ Chr length (Mb)*100

% SNPs in blocks = SNPs (n) in blocks/Number of SNP per Chr*100
