# Supplementary material for: Linkage Disequilibrium, Haplotype Block Structures, Effective Population Size and Genome-Wide Signatures of Selection of Two Conservation Herds of the South African Nguni Cattle
Source: Animals (Basel). 2022 Aug 19;12(16):2133. doi: 10.3390/ani12162133 (PMC9405234; doi:10.3390/ani12162133)
Supplement: Supplementary file 1 [file animals-12-02133-s001.zip › Table S3 - Additional file S3.pdf]

Table S3: Kokstadt haplotype block summary per chromosome (chr)

Chr	Chr length (Mb)	SNP(n) per Chr	Mean Block length (Mb)	Blocks (n)	Block coverage length (Mb)	% Chr block coverage	SNPs (n) in blocks	% SNPs in blocks	SNP average per Chr
1	158.53	41219	0.02±0.03	4129	90.59	57.14	28136	68.26	6.81
2	136.23	35798	0.02±0.03	3483	78.88	57.90	24285	67.84	6.97
3	121.01	31791	0.02±0.03	3174	67.71	55.95	21158	66.55	6.67
4	120	31341	0.02±0.03	3159	66.16	55.13	20374	65.01	6.45
5	120.09	30506	0.02±0.04	3033	70.64	58.82	20901	68.51	6.89
6	117.81	30591	0.02±0.04	3053	70.25	59.63	21310	69.66	6.98
7	110.68	29946	0.02±0.04	3021	63.56	57.43	20067	67.01	6.64
8	113.32	29568	0.02±0.03	2934	61.90	54.62	19142	64.74	6.52
9	105.45	27268	0.02±0.03	2737	58.93	55.88	18225	66.84	6.66
10	103.31	26932	0.02±0.03	2786	54.69	52.94	17494	64.96	6.28
11	106.98	27937	0.02±0.03	2788	60.83	56.86	19091	68.34	6.85
12	87.22	23121	0.02±0.04	2379	47.79	54.79	15106	65.33	6.35
13	83.47	20506	0.02±0.03	2039	47.07	56.39	13255	64.64	6.50
14	82.4	22065	0.02±0.03	2254	46.00	55.83	14387	65.20	6.38
15	85.01	21978	0.02±0.03	2263	44.78	52.68	14205	64.63	6.28
16	81.01	21470	0.02±0.03	2145	46.65	57.59	14773	68.81	6.87
17	73.17	19328	0.02±0.03	2064	40.26	55.02	13028	67.40	6.31
18	65.82	17323	0.02±0.03	1918	33.84	51.41	11424	65.95	5.96
19	63.45	16469	0.02±0.03	1772	33.03	52.06	10590	64.30	5.98
20	71.97	18331	0.02±0.03	1835	40.01	55.59	12162	66.35	6.63
21	69.86	19009	0.02±0.04	1839	39.91	57.13	12652	66.56	6.88
22	60.77	16231	0.02±0.03	1685	31.53	51.88	10677	65.78	6.34
23	52.5	13791	0.02±0.03	1527	23.67	45.09	8138	59.01	5.33
24	62.32	17019	0.02±0.02	1754	32.81	52.65	11131	65.40	6.35
25	42.35	11390	0.02±0.02	1192	21.00	49.59	6958	61.09	5.84
26	51.99	13837	0.02±0.03	1474	25.50	49.05	8572	61.95	5.82
27	45.61	11384	0.02±0.03	1200	22.00	48.24	7150	62.81	5.96
28	45.94	11492	0.02±0.02	1245	21.03	45.78	7153	62.24	5.76
29	51.1	12676	0.02±0.03	1355	26.42	51.70	7918	62.46	5.84
**Total**	**2489.37**	**650317**	**0.02±0.03**	**66237**	**1367.42**	**53.96**	**429462**	**65.44**	**6.48**
