# Supplementary material for: Linkage Disequilibrium, Haplotype Block Structures, Effective Population Size and Genome-Wide Signatures of Selection of Two Conservation Herds of the South African Nguni Cattle
Source: Animals (Basel). 2022 Aug 19;12(16):2133. doi: 10.3390/ani12162133 (PMC9405234; doi:10.3390/ani12162133)
Supplement: Supplementary file 1 [file animals-12-02133-s001.zip › Table S5 - Additional file S5.pdf]

Table S3: Bartlow and Kokstadt haploblock statistics per length category

Haploblock length categories	Haploblocks (n)	Mean (Kb)	SD	Median (Kb)	Min (Kb)	Max (Kb)
			**Bartlow population**			
0 – 10 kb	38448	4.37	2.66	3.98	0.001	9.999
10 – 25 kb	19999	16.18	4.23	15.58	10.00	25.00
25 – 50 kb	11384	35.16	7.05	34.17	25.00	50.00
50 – 100 kb	5606	67.94	13.49	65.16	50.01	99.99
100 – 150 kb	1146	119.60	13.95	117.50	100.10	149.90
150 – 200 kb	417	170.40	13.99	168.80	150.00	199.80
200 – 500 kb	283	258.60	59.08	238.80	200.00	487.00
500 – 750 kb	12	586.50	61.29	572.20	512.90	689.0
750 – 1000 kb	10	882.40	82.46	892.20	776.30	999.00
**Total**	**77305**	**237,91**	**28,69**	**234,27**	**202,70**	**301,08**
			**Kokstadt population**			
**Haploblock length categories**	**Haploblocks (n)**	**Mean (Kb)**	**SD**	**Median (Kb)**	**Min (Kb)**	**Max (Kb)**
0 – 10 Kb	32918	4.36	2.67	3.95	0.001	9.999
10 – 25 Kb	16704	16.24	4.24	15.62	10.00	25.00
25 – 50 Kb	10039	35.22	7.05	34.22	25.00	50.00
50 – 100 Kb	4931	67.90	13.53	64.90	50.01	99.96
100 – 150 Kb	1025	120.40	14.33	118.20	100.00	150.00
150 – 200 Kb	349	171.20	4.59	169.90	150.20	199.90
200 – 500 kb	248	264.70	67.44	240.60	200.40	498.00
500 – 750 kb	19	621.80	85.52	645.10	503.50	743.70
750 – 1000 kb	4	901.90	72.15	891.10	825.90	999.40
**Total**	**66237**	**244,86**	**30,17**	**242,62**	**207,22**	**308,44**
