# Supplementary material for: Linkage Disequilibrium, Haplotype Block Structures, Effective Population Size and Genome-Wide Signatures of Selection of Two Conservation Herds of the South African Nguni Cattle
Source: Animals (Basel). 2022 Aug 19;12(16):2133. doi: 10.3390/ani12162133 (PMC9405234; doi:10.3390/ani12162133)
Supplement: Supplementary file 1 [file animals-12-02133-s001.zip › Table S9 - Additional file S9.pdf]

**Chromosome**	**Genes**
7 & 8	*SLC4A9*, ENSBTAG00000054857, RF00026, ENSBTAG00000010871, *EIF4EBP3*, *SRA1*, *APBB3*, *SLC35A4*, *TMCO6*, *NDUFA2*, *IK*, *DND1*, *HARS*, *HARS2*, *ZMAT2*, *RF00006*, *PCDHA2*, ENSBTAG00000049097, ENSBTAG00000052107, *PCDHA4*, ENSBTAG00000049759, ENSBTAG00000015439, ENSBTAG00000049214, ENSBTAG00000054587, ENSBTAG00000054506, ENSBTAG00000052605, ENSBTAG00000048510, ENSBTAG00000051344, ENSBTAG00000054285, *PCDHA13*, ENSBTAG00000053013, *PCDHAC2*, ENSBTAG00000053988, *PCDHB1*, ENSBTAG00000053160, ENSBTAG00000049493, ENSBTAG00000030474, ENSBTAG00000054343, ENSBTAG00000050840, ENSBTAG00000049316, *SLC25A2*, *PCDHGA3*, ENSBTAG00000048645, *PCDHGA5*, *PCDHGC3*, ENSBTAG00000053294, ENSBTAG00000053294, ENSBTAG00000054576, ENSBTAG00000055200, ENSBTAG00000048933, *PCDHGC4* and ENSBTAG00000030499
