# Supplementary figures and images for: Linkage Disequilibrium, Haplotype Block Structures, Effective Population Size and Genome-Wide Signatures of Selection of Two Conservation Herds of the South African Nguni Cattle
Source: Animals (Basel). 2022 Aug 19;12(16):2133. doi: 10.3390/ani12162133 (PMC9405234; doi:10.3390/ani12162133)

Bartlow\_ihs

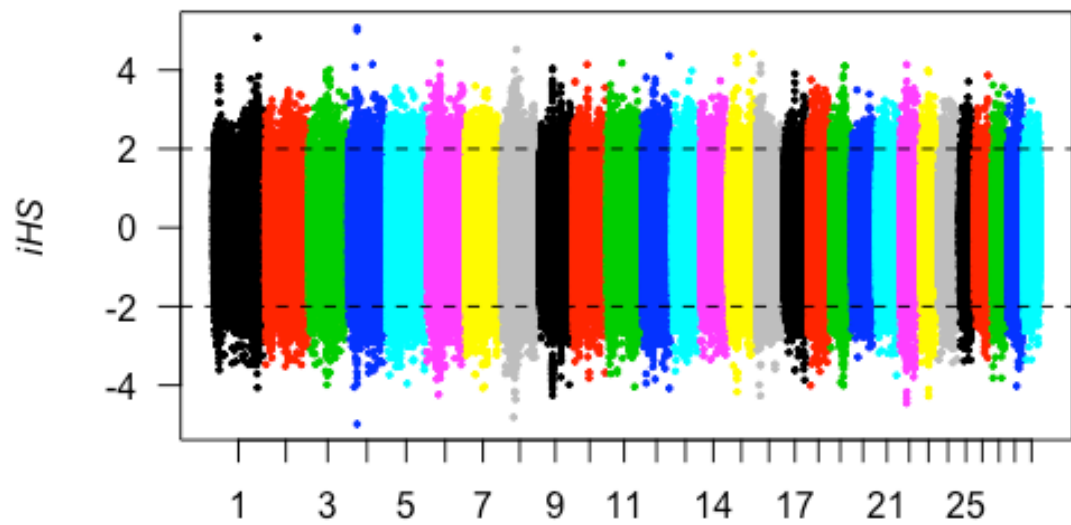

Kokstadt\_ihs

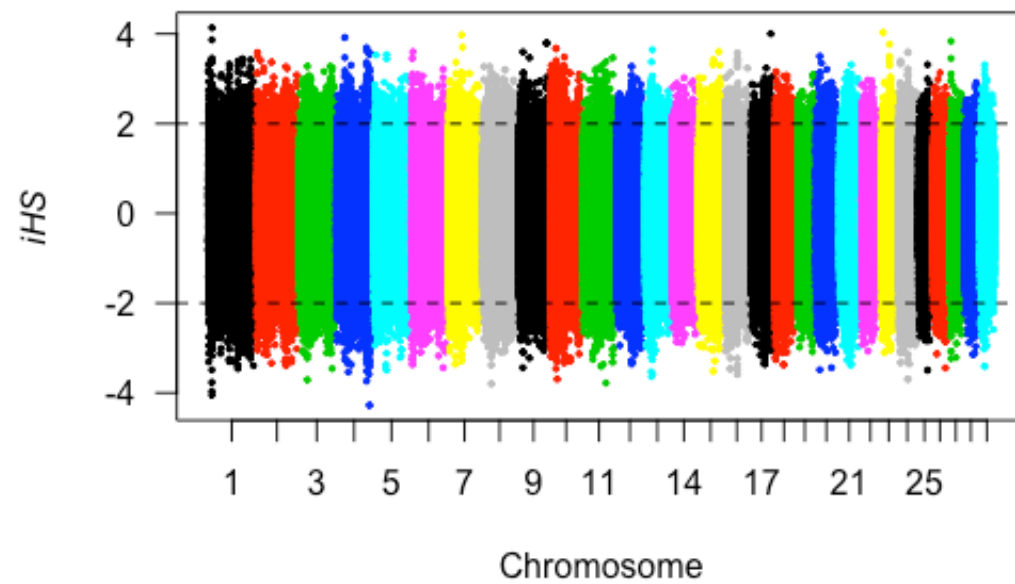

Chromosor

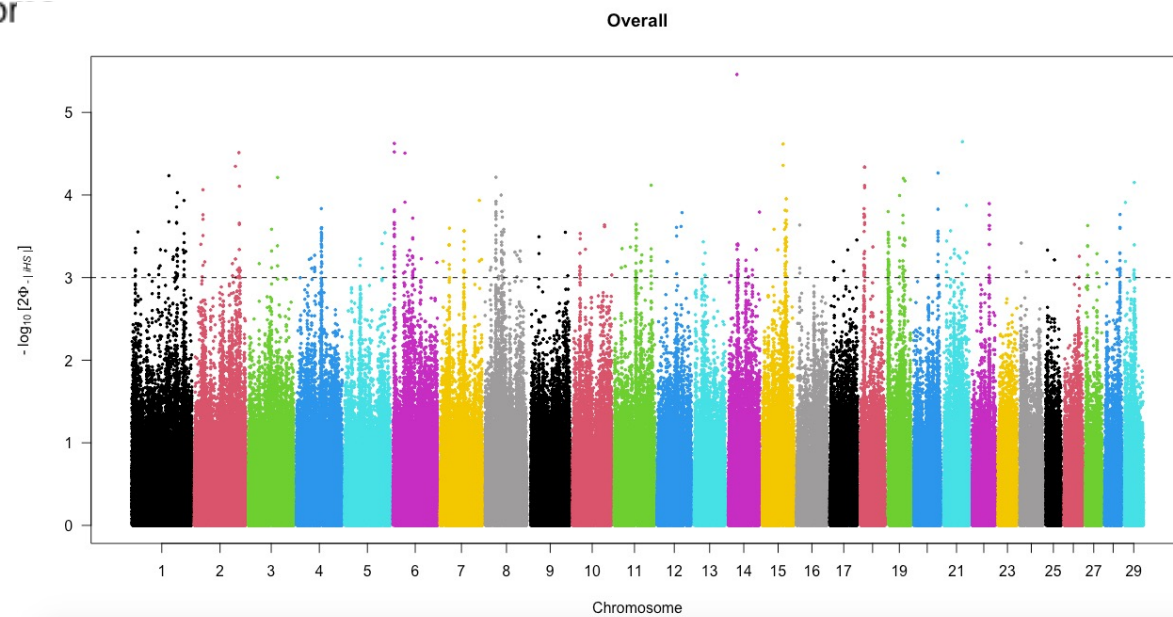

Supplement: Supplementary file 1 [file animals-12-02133-s001.zip › Figure S8 Supplementary File S8.pdf]
